# Walnut Septum-Derived Aqueous Extract Alleviates Colitis Through Modulation of Gut Metabolism and Inflammatory Signaling

**DOI:** 10.3390/foods15111866

**Published:** 2026-05-25

**Authors:** Beier Jiang, Yu Wan, Lina Liu, Jiajun Cheng, Tianjiao Min, Xinlong Gao, Zicheng Yu, Li Ma, Ying He

**Affiliations:** 1 Navy Medical Centre, Naval Medical University, Shanghai 200433, China; 2 College of Food Science and Technology, Shanghai Ocean University, Shanghai 201306, China

**Keywords:** ulcerative colitis, *Diaphragma Juglandis* Fructus, JAK1-STAT3, multi-omic, metabolic reprogramming

## Abstract

The aqueous extract of *Diaphragma Juglandis* Fructus (AED), a by-product of *Juglans regia* L., represents a promising food-derived functional ingredient with potential benefits for intestinal health. This study evaluated the anti-colitis effects of AED and explored its underlying mechanisms using LPS-stimulated RAW264.7 macrophages and a DSS-induced colitis mouse model. In DSS-induced colitis in mice, AED at 10 μg/mL suppressed pro-inflammatory cytokine production and inhibited JAK1/STAT3 signaling. In DSS-induced colitis in mice, AED at 600 mg/kg for 7 days mitigated DSS-induced colonic injury, restored tight junction proteins, and improved epithelial barrier integrity. Integrated transcriptomic and metabolomic analyses identified AED-associated alterations in arginine-polyamine and taurine-hypotaurine metabolism, while network pharmacology and molecular docking suggested angiotensin-converting enzyme (ACE) and von Willebrand factor (VWF) as candidate functional targets for further investigation. Collectively, these findings indicate that AED exerts anti-colitis effects in association with coordinated changes in inflammatory signaling, metabolic pathways, and barrier-related markers, supporting its potential as a food-derived functional ingredient candidate for ulcerative colitis management.

## 1. Introduction

Ulcerative colitis (UC) is a chronic, relapsing inflammatory bowel disease characterized by diarrhea, abdominal pain, and rectal bleeding, which substantially impairs patients’ physical and psychological well-being [1,2]. Its pathogenesis involves a complex interplay among environmental factors, host genetic susceptibility [3], immune dysregulation [4,5], and gut microbiota imbalance [6], ultimately leading to epithelial barrier dysfunction and sustained mucosal immune activation. Although current therapeutic strategies, including aminosalicylates, glucocorticoids, and immunomodulators, can alleviate intestinal inflammation, their clinical efficacy is often limited by heterogeneous therapeutic responses, dose-dependent adverse effects, and concerns regarding long-term safety [2,7,8]. The continuously rising global incidence of UC highlights an urgent need for novel therapeutic approaches that provide durable inflammatory control while promoting epithelial barrier restoration with minimal systemic toxicity.

The Janus kinase (JAK)–signal transducer and activator of transcription 3 (STAT3) signaling pathway plays a pivotal role in UC pathogenesis by orchestrating mucosal inflammation and epithelial barrier disruption [9]. Upon activation by pro-inflammatory cytokines such as interleukin-6 (IL-6) and IL-23, JAK–STAT3 signaling promotes Th17 cell differentiation, immune cell recruitment, tissue remodeling, and destabilization of tight junctions [10,11,12]. Elevated STAT3 phosphorylation has been consistently observed in the colonic mucosa of UC patients and correlates with disease severity. Accordingly, pharmacological inhibition of JAK–STAT3 signaling has emerged as a promising therapeutic strategy. JAK inhibitors, including tofacitinib and upadacitinib, have been approved for moderate-to-severe UC and demonstrate efficacy in both induction and maintenance of remission by attenuating cytokine-driven inflammation [13,14]. However, these agents are associated with clinically relevant safety concerns, such as increased susceptibility to infections, hyperlipidemia, venous thromboembolism, and cardiovascular events, necessitating careful patient stratification and long-term monitoring [15,16].

In addition to inflammatory signaling, metabolic dysregulation is increasingly recognized as an important feature of UC pathogenesis. Among the relevant pathways, arginine/polyamine-related metabolism is associated with immune regulation, mucosal repair, and epithelial proliferation, whereas taurine/hypotaurine metabolism is involved in antioxidant defense and intestinal inflammatory homeostasis. Disturbance of these pathways may contribute to persistent mucosal injury and barrier dysfunction, suggesting their potential relevance to colitis intervention [17].

Dietary bioactive components derived from plant-based foods are increasingly recognized for their capacity to support intestinal health through multi-target regulatory mechanisms. Growing evidence suggests that food-derived phytochemicals and macromolecules can modulate inflammatory signaling pathways, rebalance immune homeostasis, and reinforce epithelial barrier integrity, thereby contributing to the maintenance of gut function [18,19]. Unlike single-target pharmacological agents, food-derived compounds often exert coordinated and mild regulatory effects, making them particularly suitable for long-term dietary management of chronic intestinal disorders [20]. In experimental models of UC, accumulating studies demonstrate that plant-based functional components alleviate intestinal inflammation by attenuating oxidative stress, regulating cytokine networks, and preserving mucosal barrier structure. For example, polyphenols derived from green pea hulls significantly ameliorate dextran sulfate sodium (DSS)-induced colitis by suppressing colonic cytokine release [21], while cherry polyphenols reduce lipid peroxidation and enhance endogenous antioxidant defenses [22]. In addition, polysaccharides isolated from various plant sources have been shown to strengthen tight junction integrity and improve epithelial barrier function, further supporting the therapeutic relevance of plant-derived macromolecules in intestinal inflammation.

*Diaphragma Juglandis* Fructus (DJF), commonly known as walnut septum, is an underutilized agro-industrial by-product of *Juglans regia* L., which contributes to environmental burdens. While traditionally consumed or utilized in dietary preparations, DJF remains underused, highlighting the potential for its valorization as a sustainable resource. Recent literature has reported DJF as a promising raw material for extraction and further utilization. Various extraction approaches have been applied to prepare DJF-derived extracts or fractions, laying a foundation for their biological investigation [23,24]. Phytochemical investigations have revealed that DJF is enriched in polysaccharides, flavonoids, and polyphenols [24], which exhibit documented anti-inflammatory [25], antibacterial [26], and antioxidant activities [27]. Moreover, DJF extracts have been reported to suppress nitric oxide (NO) production and scavenge reactive oxygen species [28]. Despite these bioactivities, systematic chemical standardization and mechanistic evaluation of DJF in UC remain insufficient. In particular, whether DJF modulates metabolic remodeling and epithelial barrier repair via JAK1-STAT3-associated signaling pathways remains unclear.

Beyond inflammatory signaling and epithelial barrier disruption, vascular dysfunction may contribute to UC progression. Angiotensin-converting enzyme (ACE) is involved in inflammation and oxidative stress, while von Willebrand factor (VWF) is a marker of endothelial activation and vascular injury, suggesting their potential relevance in colitis [29,30]. Given the multi-component nature of AED, network pharmacology and molecular docking were employed to predict potential targets, and molecular simulations were used to assess compound–target interactions.

In the present study, we aimed to comprehensively evaluate the protective efficacy and underlying molecular mechanisms of the aqueous extract of *Diaphragma Juglandis* Fructus (AED) in experimental colitis models. The chemical profile of AED was standardized using UPLC-MS/MS to facilitate its quality control and potential food application. Cellular and animal experiments were conducted to assess its anti-inflammatory and barrier-supporting activities. Furthermore, integrated transcriptomic and untargeted metabolomic analyses were performed to identify AED-associated metabolic alterations, including arginine/polyamine-related metabolism and taurine/hypotaurine metabolism, and to explore their associations with JAK1/STAT3 signaling and tight junction proteins. Collectively, this work provides multi-level evidence that AED is associated with beneficial changes in inflammatory responses, barrier-related markers, and metabolic pathways in experimental colitis, supporting its further evaluation as a standardized food-derived functional ingredient for intestinal health management.

## 2. Materials and Methods

### 2.1. Preparation of AED

#### 2.1.1. Aqueous Extract Preparation

Dried DJF (1000 g) was milled (fine powder) and extracted with distilled water (10 L; 1:10 *w*/*v*). The suspension was stirred at 25 °C for 2 h, then refluxed at 100 °C for 1 h. After vacuum filtration to remove insolubles, the filtrate was concentrated under reduced pressure at 40 °C to ~200 mL using a rotary evaporator (EYELA, Tokyo, Japan), then centrifuged using a 5418R refrigerated centrifuge (Eppendorf, Hamburg, Germany), snap-frozen at −70 °C for 6 h, and lyophilized at −50 °C for 48 h to yield AED [31]. The extraction yield of AED was approximately 9% in the present study, consistent with our previous findings obtained using the same source material and aqueous extraction procedure. AED was stored in airtight amber containers at 4 °C until use. For biological experiments, the aqueous working solution was freshly prepared before use.

#### 2.1.2. Chemical Profiling of AED by UPLC-MS/MS

AED, prepared as described in Section 2.1.1, was profiled on a mass spectrometry platform (Bruker Daltonics, Bremen, Germany), with chromatographic separation performed using an Agilent 1290 Infinity II system (Agilent, Santa Clara, CA, USA). Analytical standards (≥98%, Sigma-Aldrich, St. Louis, MO, USA) were used for confirmation. AED (1 mg/mL in HPLC-grade methanol) was vortexed for 30 s and centrifuged at 10,000 rpm for 5 min. The supernatant was mixed 1:1 with DHB (10 mg mL^−1^, 70:30 acetonitrile: water, 0.1% TFA), and 1 µL was spotted per target and air-dried. Working conditions: negative-ion, reflectron mode; *m*/*z* range of 50–2000; 1 kHz laser. External calibration with Peptide Calibration Standard II (Bruker Daltonics, Bremen, Germany) achieved mass accuracy ≤ 5 ppm. For selected ions, TOF/TOF MS/MS spectra were acquired to confirm identities against standards. Spectra were acquired with FlexControl 3.0 and processed in FlexAnalysis 3.4. Each sample was spotted and measured in triplicate, and peaks were only accepted if they were observed across replicates, had a signal-to-noise ratio above the method threshold, and had a mass error ≤ 5 ppm relative to the corresponding standard.

### 2.2. Cell Experiments

#### 2.2.1. Cell Viability Assay

RAW264.7 cells(Procell, Wuhan, China) were seeded in 96-well plates at 7 × 10^3^ cells/well and incubated overnight at 37 °C with 5% CO_2_. Cell viability was assessed after treatment with AED (0.1, 0.5, 1, 5, 10, and 50 μg/mL) for 24 h. In parallel, the effect of LPS (Sigma-Aldrich, St. Louis, MO, USA) (100 ng/mL) on cell viability was also examined under the same conditions. Vehicle controls were included. Following treatment, 10% (*v*/*v*) CCK-8 (Beyotime, Shanghai, China) in fresh DMEM was added (100 µL/well), and the plate was incubated for 2 h, and absorbance was measured at 450 nm. All experiments were performed in triplicate and repeated at least three times.

#### 2.2.2. Secretion of NO and Inflammatory Cytokines

RAW264.7 cells were seeded in 96-well plates at 3 × 10^4^ cells/well and incubated overnight (37 °C, 5% CO_2_). Cells were pre-treated with AED (0.1, 0.5, 1, 5, 10, 50 µg/mL) for 1 h, then stimulated with LPS (100 ng/mL) for 24 h (AED maintained during stimulation). NO production in culture supernatants was quantified using the Griess assay kit (Beyotime, Shanghai, China) according to the manufacturer’s instructions, and nitrite concentration was calculated from a sodium nitrite standard curve (0–100 μM). Absorbance was measured at 540–550 nm.

The concentrations of IL-6, IL-1β, and TNF-α in the supernatants were determined by ELISA (hnybio, Changsha, China) according to the manufacturer’s protocol.

#### 2.2.3. RNA Isolation and Quantitative Real-Time PCR Assay

RAW264.7 cells were seeded in 6-well plates at 5 × 10^5^ cells/mL and treated with AED (1, 5, and 10 μg/mL), followed by stimulation with LPS (100 ng/mL) for 24 h. Total RNA was extracted using RNAiso Plus (TaKaRa, Beijing, China) and reverse-transcribed into cDNA using PrimeScript™ RT Master Mix. Quantitative real-time PCR was performed using SYBR^®^ Premix Ex Taq™ on a real-time PCR system according to the manufacturer’s protocol. RT-qPCR was used to determine the mRNA expression of iNOS, IL-6, IL-1β, and TNF-α, which were selected as representative markers of LPS-induced inflammatory responses. GAPDH was used as the internal control, and the specific primer sequences used in this study are listed in Supplementary Table S1.

#### 2.2.4. Western Blot

RAW264.7 cells were treated with LPS in the presence or absence of AED as described in Section 2.2.3. Cells were washed with ice-cold PBS and lysed in RIPA buffer (Beyotime, Shanghai, China) supplemented with protease and phosphatase inhibitors. Lysates were clarified by centrifugation (12,000× *g*, 10 min, 4 °C), and protein concentrations were determined using a BCA assay (Beyotime, Shanghai, China).

Equal amounts of protein (20–30 μg) were separated by 10% SDS–PAGE and transferred onto PVDF membranes (0.45 μm). Membranes were blocked with 5% non-fat milk in TBST and incubated overnight at 4 °C with primary antibodies against iNOS, COX-2, JAK1, p-JAK1 (Tyr1034/1035), STAT3, p-STAT3 (Tyr705), and β-actin. After incubation with HRP-conjugated secondary antibodies, immunoreactive bands were visualized using enhanced chemiluminescence. Band intensities were quantified using ImageJ software (version 1.54). Phosphorylation levels were expressed as the ratio of phosphorylated to total protein and normalized to the loading control.

### 2.3. Animal Experiment

#### 2.3.1. Animals and Experimental Design

All animal procedures were approved by the Animal Experiments Committee of the PLA Naval Medical Center (Approval ID: 2025H007) and complied with institutional guidelines. Fifty male C57BL/6 mice (SPF Biotechnology, Beijing, China); 6–8 weeks old and weighing 18–20 g) were acclimated for 3 days under SPF conditions (20 °C, 12 h light/dark cycle) with ad libitum access to chow (Suzhou Shuangshi Feed Technology, Suzhou, China) and water. The sample size and animal sex selection were consistent with the standardized DSS-induced colitis protocol and related studies [32]. Animals were randomly assigned to five groups (n = 10/group) using a random number table: Control, DSS (3% *w*/*v*), sulfasalazine (SASP; 300 mg/kg), high-dose AED (HAED; 600 mg/kg), and low-dose AED (LAED; 200 mg/kg). For each group, mice were housed in two cages, with five mice per cage.

Acute colitis was induced with 3.0% DSS (MP Biomedicals, Santa Ana, CA, USA) in drinking water for 7 days. Treatments (SASP or AED) were administered once daily by oral gavage using saline as the vehicle throughout DSS exposure, while the Control and DSS groups received an equal volume of saline (0.2 mL per mouse). Outcome assessment and data analysis were performed blinded to group allocation whenever possible. The disease activity index (DAI) was calculated based on three parameters: body weight loss, stool consistency, and fecal blood. Each parameter was scored as follows: body weight loss (0 = no loss, 1 = 1–5%, 2 = 5–10%, 3 = 10–20%, 4 = >20%), stool consistency (0 = normal, 1 = loose, 2 = diarrhea), and fecal blood (0 = none, 1 = traces, 2 = moderate, 3 = severe) [33]. Body weight was recorded for each mouse, whereas stool consistency and fecal blood were assessed at the cage level because fecal samples could not be assigned to individual mice. Therefore, DAI was calculated and analyzed at the cage level, with each cage considered one experimental unit for DAI analysis (n = 2 cages/group). Mice were monitored daily for body weight, stool consistency, fecal blood, and general condition throughout the experiment. In the DSS-induced acute colitis model, body weight loss, loose stool, and fecal blood are expected indicators of disease induction and were included in DAI scoring; therefore, humane intervention was considered based on overall severity and general condition rather than on these signs alone. Animals would have been humanely euthanized if severe distress or marked deterioration in general condition had occurred. No unexpected adverse events occurred, and no animals required early euthanasia or removal from the study. On day 7, mice were euthanized, and colons were excised, and their lengths were measured. After collection, colons were flushed with sterile PBS at 4 °C to remove fecal contents, and then snap-frozen in liquid nitrogen and stored at −80 °C for subsequent analyses.

#### 2.3.2. Histopathology and Immunohistochemistry

Distal colon tissues were fixed in 10% neutral buffered formalin, paraffin-embedded, and sectioned at 4 μm for histopathological evaluation. Hematoxylin and eosin (HE) staining was performed using standard protocols.

For IHC, distal colon tissue sections from four mice per group were deparaffinized and rehydrated, followed by heat-induced antigen retrieval in citrate buffer (pH 6.0). Endogenous peroxidase activity was quenched with H_2_O_2_, and nonspecific binding was blocked with normal serum. Sections were incubated overnight at 4 °C with primary antibodies against ZO-1, Occludin, Claudin-1, iNOS, COX-2, and p-STAT3, followed by incubation with HRP-conjugated secondary antibodies. Images were acquired under a light microscope and analyzed using ImageJ software (version 1.54).

#### 2.3.3. RNA Isolation and Quantitative Real-Time PCR Assay

Total RNA was isolated from colon tissues, and quantitative real-time PCR was performed as described in Section 2.2.3 to assess the relative mRNA expression levels of iNOS, IL-6, IL-1β, and TNF-α.

#### 2.3.4. Transcriptional Profiling

Total RNA from distal colon tissues was extracted using a commercial kit and treated with DNase. RNA quality was confirmed by spectrophotometry (A260/280 = 1.9–2.1). Poly(A)+ libraries were prepared and sequenced on an Illumina NovaSeq platform (paired-end, 150 bp) by Novogene (Beijing, China).

Reads were quality-filtered and aligned to the mouse genome (GRCm39, Ensembl 104) using STAR v2.7.10a. Gene counts were generated with featureCounts v2.0.3, and differential expression was analyzed using DESeq2 with group as the design factor (Control, DSS, HAED). Genes with |log_2_FC| ≥ 1.5 and adjusted *p* < 0.05 were defined as differentially expressed genes (DEGs). Functional enrichment (GO and KEGG) was performed using clusterProfiler v4.6.0 with Benjamini–Hochberg correction (q < 0.05). Both ORA (cutoff-based) and GSEA (ranked by signed Wald statistic) were conducted. Differential expression was analyzed using DESeq2. *p* values were adjusted using the Benjamini–Hochberg method to control the false discovery rate (FDR < 0.05). Effect sizes are presented as log_2_-fold changes, and 95% confidence intervals were calculated as log_2_-fold change ± 1.96 × standard error.

#### 2.3.5. Metabolomics Profiling

Untargeted LC-MS metabolomics of colon samples was performed by Novogene (Beijing, China). QC samples were injected periodically, and features with QC RSD > 30% or detected in blanks were removed. Data were normalized, log_2_-transformed, and Pareto-scaled. Differential metabolites were identified using univariate tests with FDR correction (q < 0.05). PCA and PLS-DA were used for visualization, with cross-validation and permutation testing to assess model robustness. Pathway enrichment was performed using MetaboAnalyst 6.0 with KEGG identifiers. Transcriptomic and metabolomic pathways were integrated based on KEGG IDs.

#### 2.3.6. Protein and Ligand Preparation

Crystal structures of ACE (PDB: 3L3N) and von VWF (PDB: 3HXQ) were obtained from the PDB and prepared using UCSF Chimera v1.17 Chimera by removing non-protein components and adding hydrogens. Ligand structures were downloaded from PubChem, optimized using MMFF94 (via Open Babel), and converted to PDBQT format using Open Babel v3.1.1.

#### 2.3.7. Docking Protocol and Analysis

Molecular docking was performed using AutoDock 4.2.6 with grid boxes covering the predicted binding sites. The Lamarckian Genetic Algorithm was used with 50 runs per ligand. Docked poses were ranked by predicted binding energy, and the lowest-energy conformation was selected for interaction analysis using Chimera v1.17 and PyMOL v2.5.

#### 2.3.8. Target Identification

Putative targets were identified by literature mining and reverse screening using PharmMapper (http://www.lilab-ecust.cn/pharmmapper/, accessed on 30 April 2026), and further cross-validated using SwissTargetPrediction (http://www.swisstargetprediction.ch/, accessed on 30 April 2026) and SEA (https://sea.bkslab.org/, accessed on 30 April 2026). Targets supported by at least two databases were retained. ACE and VWF were prioritized for docking and molecular dynamics analyses based on biological relevance.

#### 2.3.9. Molecular Dynamics Simulation

MD simulations were performed using GROMACS v2023.2 with the AMBER99SB-ILDN force field. Systems were solvated with TIP3P water, neutralized, energy-minimized, and equilibrated, followed by 200 ns production runs. Structural stability was evaluated using RMSD, RMSF, Rg, and hydrogen bond occupancy in GROMACS and MDAnalysis.

#### 2.3.10. Gene–Metabolite Correlation Network Construction

A bipartite undirected network was constructed in which metabolite nodes and gene nodes are connected by edges representing significant Spearman correlations (|ρ| > 0.7, *p* < 0.05). This threshold was selected to retain only strong, statistically supported associations while maintaining a tractable network topology. Network construction and centrality analysis were performed using NetworkX v3.1 (Python). For each node, degree centrality (C_D) and betweenness centrality (C_B) were computed, and a composite hub centrality score was defined as H = C_D + C_B. Nodes with H ≥ 70th percentile of all hub scores were designated as hub nodes. Network layout was generated using the shell algorithm (nx.shell_layout), with the ten highest-H nodes positioned in the inner ring and all remaining nodes distributed uniformly on the outer ring. Edge width and opacity were scaled linearly with |ρ|. Node size was scaled linearly with H. Node color encodes log_2_FC using a diverging RdBu_r colormap normalized to the range [−3, +3] with a two-slope normalization centered at zero (matplotlib.colors.TwoSlopeNorm).

#### 2.3.11. SVD-Based Multi-Omics Latent Factor Analysis

To identify shared axes of variation between the metabolome and transcriptome, a joint feature matrix was constructed as follows. For metabolomics, log_2_-transformed intensities (log_2_[x + 1]) of the top 50 metabolites (ranked by VIP score) were z-score normalized across samples for each feature independently. For transcriptomics, CPM-normalized expression values of the top 50 DEGs (|log_2_FC| > 1, *p* < 0.05, ranked by log_2_FC) were similarly z-score normalized. The two normalized matrices (each dimension: 5 samples × 50 features) were horizontally concatenated to yield a 5 × 100 integrated matrix. Truncated SVD was applied to this matrix using scikit-learn (TruncatedSVD, n_components = 3, random_state = 42) to extract three latent factors. The proportion of total variance explained by each factor was computed from the singular values. Feature loadings on Factors 1 and 2 were visualized in a biplot, in which the top 14 metabolites and top 10 genes—selected based on Euclidean distance from the origin in the two-dimensional loading space—were annotated. All analyses were performed in Python 3.11 using NumPy v1.24, pandas v2.0, scikit-learn v1.3, matplotlib v3.7, and adjustText v0.8.

### 2.4. Statistical Analysis

All data are presented as means ± standard deviations (SDs). Statistical analyses were performed using GraphPad Prism (version 9.5.1). Before analysis, the normality of data distribution and homogeneity of variances were validated using the Shapiro–Wilk test and Levene’s test, respectively. Comparisons among multiple groups were conducted using one-way analysis of variance (ANOVA), followed by Tukey’s post hoc test when variances were homogeneous or Dunnett’s T3 test when variances were unequal. For body weight and DAI scores involving multiple time points, two-way repeated-measures ANOVA followed by Tukey’s post hoc test was used. A two-sided *p* value < 0.05 was considered statistically significant.

## 3. Results

### 3.1. Identification of Significant Components in AED

The chemical profile of AED was characterized using UPLC-MS/MS for quantitative marker analysis and LC-QTOF-MS for untargeted profiling. Analytical stability was monitored using pooled QC samples, and features with QC RSD > 30% were excluded, indicating acceptable analytical reproducibility (Supplementary Figure S1 and Supplementary Table S2). Table S2 lists the representative molecular markers detected in AED, including quercitrin, C17-sphinganine, 6-methoxyflavonol, succinic acid, and catechin, which were used for chemical annotation and pathway association.

A total of 31 constituents were putatively annotated at MSI confidence levels 2–3 across positive- and negative-ion modes. The identified compounds were mainly classified as alkaloid- and peptide-like metabolites, with a smaller proportion of phenolic compounds (Supplementary Table S3). These results indicate that AED contains chemically diverse constituents suitable for subsequent pharmacological and mechanistic investigations.

### 3.2. Effect of AED on LPS-Induced Inflammatory Response in RAW264.7 Cells

#### 3.2.1. Effect of AED on the Expression of Inflammatory Cytokines and Inflammation-Related Proteins

AED (<10 μg/mL) showed no cytotoxicity in RAW264.7 cells, and LPS did not significantly affect cell viability under the conditions used in this study. Based on this safety profile, AED dose-dependently inhibited LPS-induced NO production without altering basal levels, confirming its anti-inflammatory activity (Supplementary Figure S2). Building on this foundation, we further examined the regulatory effects of AED on key pro-inflammatory cytokines.

**Figure 1:**
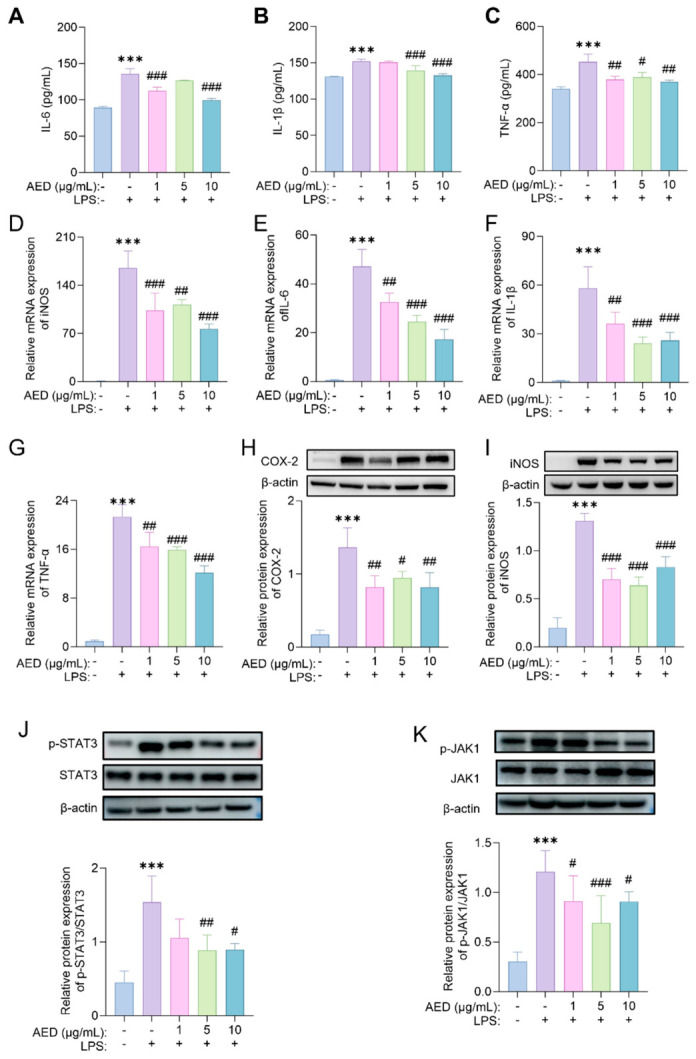
Effects of AED on inflammatory cytokine expression and the JAK1/STAT3 signaling pathway in LPS-stimulated RAW264.7 macrophages. (**A**–**C**) Secretion levels of IL-6, IL-1β, and TNF-α measured by ELISA (n = 3). (**D**–**G**) Relative mRNA expression of iNOS, IL-6, IL-1β, and TNF-α determined by RT-qPCR (n = 4). (**H**,**I**) Protein expression of iNOS and COX-2 detected by Western blot (n = 4). Data are presented as mean ± SD. (**J**,**K**) Protein expression of JAK1, p-JAK1, STAT3, and p-STAT3 in RAW264.7 cells treated with AED. Data are presented as mean ± SD (n = 4). Statistical significance was assessed by one-way ANOVA followed by Tukey’s post hoc test or Dunnett’s T3 test as appropriate. * indicates comparisons versus the Control group (*** *p* < 0.001), and # symbols denote comparisons versus the LPS group (# *p* < 0.05, ## *p* < 0.01, ### *p* < 0.001).

Activation of macrophages by LPS drives robust secretion of IL-6, IL-1β, and TNF-α [34]. RAW264.7 cells were treated with AED (1, 5, and 10 μg/mL) in the presence or absence of LPS (100 ng/mL) for 24 h, and cytokine levels in culture supernatants were measured by ELISA. LPS markedly increased IL-6, IL-1β, and TNF-α secretion, whereas AED significantly reduced LPS-induced cytokine production (Figure 1A–C). Significant inhibition was observed at 1 μg/mL, with the most significant reduction at 10 μg/mL. To further evaluate the anti-inflammatory effect of AED at the transcriptional level, RT-qPCR analysis was performed. LPS significantly upregulated the mRNA expression of iNOS, IL-6, IL-1β, and TNF-α, whereas AED treatment dose-dependently attenuated these increases (Figure 1D–G).

iNOS and COX-2 are inducible inflammatory enzymes upregulated in LPS-stimulated macrophages [34]. RAW264.7 cells were treated as above, and iNOS and COX-2 protein expression was analyzed by Western blot. LPS increased iNOS and COX-2 expression, while AED dose-dependently suppressed their LPS-induced upregulation (Figure 1H,I).

Together, these results indicate that AED attenuates LPS-induced cytokine release and reduces iNOS/COX-2 induction in RAW264.7 macrophages at non-cytotoxic concentrations (1–10 μg/mL).

#### 3.2.2. Effect of AED on JAK-STAT3 Signaling Pathway

The STAT3 signaling pathway plays a crucial role in immune responses and inflammatory processes, and the phosphorylation of JAK1 is involved in STAT3 activation [35]. To determine whether AED modulates this pathway, RAW264.7 macrophages were pre-treated with AED (1–10 μg/mL) and then stimulated with LPS (100 ng/mL) for 24 h. As shown in Figure 1J,K, LPS significantly increased p-JAK1 and p-STAT3, while total JAK1 and STAT3 levels remained unchanged. AED significantly reduced p-JAK1/JAK1 and p-STAT3/STAT3 compared with the LPS group, with the most pronounced inhibition observed at 5 μg/mL. AED alone did not significantly affect basal phosphorylation. Collectively, these results indicate that AED significantly suppresses LPS-induced JAK1-STAT3 signaling in RAW264.7 macrophages.

### 3.3. Effects of AED on DSS-Induced UC Mice

#### 3.3.1. Effect of AED on DAI and Colonic Tissue

**Figure 2:**
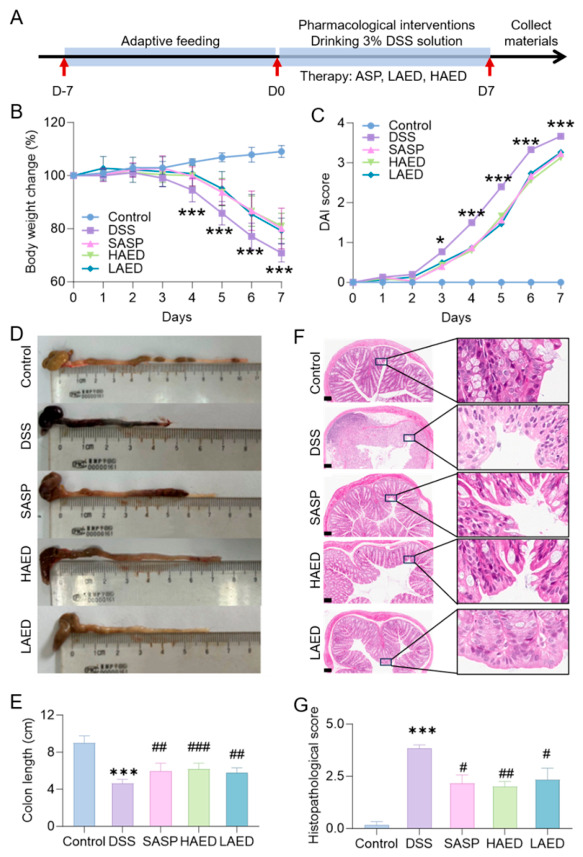
Effects of AED on clinical and histopathological features in DSS-induced colitis. (**A**) Experimental design and treatment schedule. (**B**) Changes in body weight during DSS exposure (n = 10). (**C**) Disease activity index (DAI) scores throughout the experiment. DAI was assessed at the cage level and is presented descriptively. (**D**,**E**) Representative images of colonic appearance and quantification of colon length (n = 8). (**F**,**G**) HE staining of distal colon sections showing mucosal architecture and inflammatory infiltration (magnification × 100) (n = 6). Statistical significance for (**B**) was assessed by two-way repeated-measures ANOVA, while (**E**,**G**) were analyzed by one-way ANOVA, followed by Tukey’s post hoc test or Dunnett’s T3 test as appropriate. * indicates comparisons versus the Control group (* *p* < 0.05, *** *p* < 0.001), and # symbols denote comparisons versus the DSS group (# *p* < 0.05, ## *p* < 0.01, ### *p* < 0.001).

Based on its inhibitory effects on LPS-induced inflammatory responses in RAW264.7 macrophages, AED was further evaluated in a DSS-induced acute colitis model (Figure 2A). Colitis was induced by DSS (3% *w*/*v*) for 7 d, and mice were treated once daily with AED (200 or 600 mg/kg) or SASP (300 mg/kg). DSS caused progressive body-weight loss compared with the Control group, whereas AED and SASP significantly attenuated weight loss from day 4 through day 7 (Figure 2B). Consistently, DAI, integrating body-weight loss, stool consistency, and rectal bleeding, was significantly reduced by AED and SASP (Figure 2C). DSS also induced marked colon shortening, which was partially prevented by treatment, with the high-dose AED group (600 mg/kg) showing the most significant preservation of colon length (Figure 2D,E). Histological analysis of distal colon sections further supported these findings. DSS induced mucosal edema, crypt architectural disruption, and inflammatory cell infiltration, whereas AED and SASP reduced inflammatory infiltration and improved mucosal integrity compared with the DSS group (Figure 2F,G). Collectively, AED significantly alleviated disease severity in DSS-treated mice, with greater efficacy at 600 mg/kg.

#### 3.3.2. Effect of AED on the Expression of Inflammatory Cytokines in Colon Tissues

**Figure 3:**
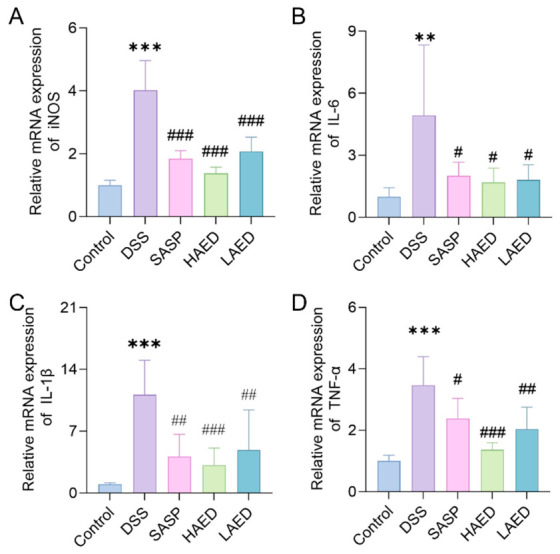
Effects of AED on inflammatory cytokine expression in colonic tissues. Relative mRNA expression levels of (**A**) iNOS, (**B**) IL-6, (**C**) IL-1β, and (**D**) TNF-α in colon tissues from DSS-induced colitis mice treated with AED. Data represent mean ± SD. (n = 6). Statistical significance was assessed by one-way ANOVA followed by Tukey’s post hoc test or Dunnett’s T3 test as appropriate. * indicates comparisons versus the Control group (** *p* < 0.01, *** *p* < 0.001), and # symbols indicate comparisons versus the DSS group (# *p* < 0.05, ## *p* < 0.01, ### *p* < 0.001).

To assess colonic inflammatory gene expression in DSS-induced colitis, the mRNA levels of iNOS, IL-6, IL-1β, and TNF-α were quantified by RT–qPCR. DSS markedly upregulated all four transcripts compared with the Control group (Figure 3A–D). Treatment with SASP or AED significantly reduced these DSS-induced increases, with the most pronounced suppression observed in the high-dose AED group (Figure 3A–D). These results indicate that AED attenuates DSS-induced colonic inflammatory responses at the transcriptional level.

#### 3.3.3. Effect of AED on the Expression of Tight Junction Proteins

**Figure 4:**
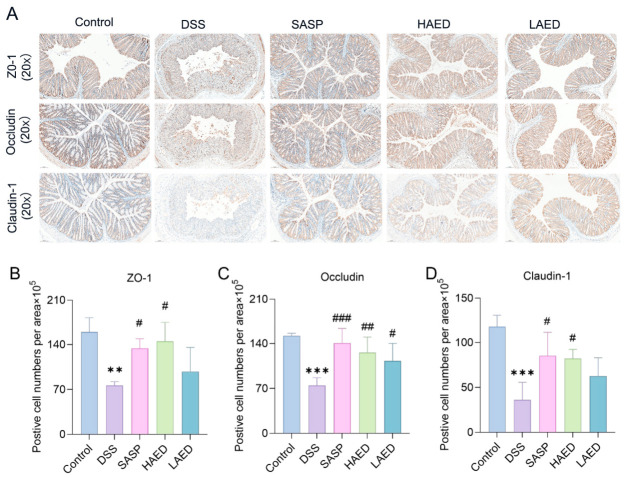
Effects of AED on tight junction protein expression in colonic tissues. (**A**) Representative immunohistochemical staining of ZO-1, Occludin, and Claudin-1 in distal colon sections. (**B**–**D**) Quantification of average optical density for ZO-1, Occludin, and Claudin-1 expression. Data are presented as mean ± SD. (n = 4). Statistical significance was assessed by one-way ANOVA followed by Tukey’s post hoc test or Dunnett’s T3 test as appropriate. * indicates comparisons versus the Control group (** *p* < 0.01, *** *p* < 0.001), and # symbols denote comparisons versus the DSS group (# *p* < 0.05, ## *p* < 0.01, ### *p* < 0.001).

Tight junction proteins are essential for epithelial barrier function and are critical determinants of intestinal mucosal integrity [36]. We assessed ZO-1, occludin, and claudin-1 in the distal colon by immunohistochemistry. Compared with the DSS group, both SASP and AED increased the intensity and continuity of junctional staining for all three proteins, accompanied by reduced diffuse cytoplasmic staining (Figure 4A–D). These findings reflect an improvement in epithelial barrier structure. Importantly, no quantitative analysis of protein expression levels was performed, as these markers were used solely to evaluate barrier integrity.

### 3.4. Transcriptomics Analysis of Effects of the AED Intervention in DSS-Induced Colitis Mice

**Figure 5:**
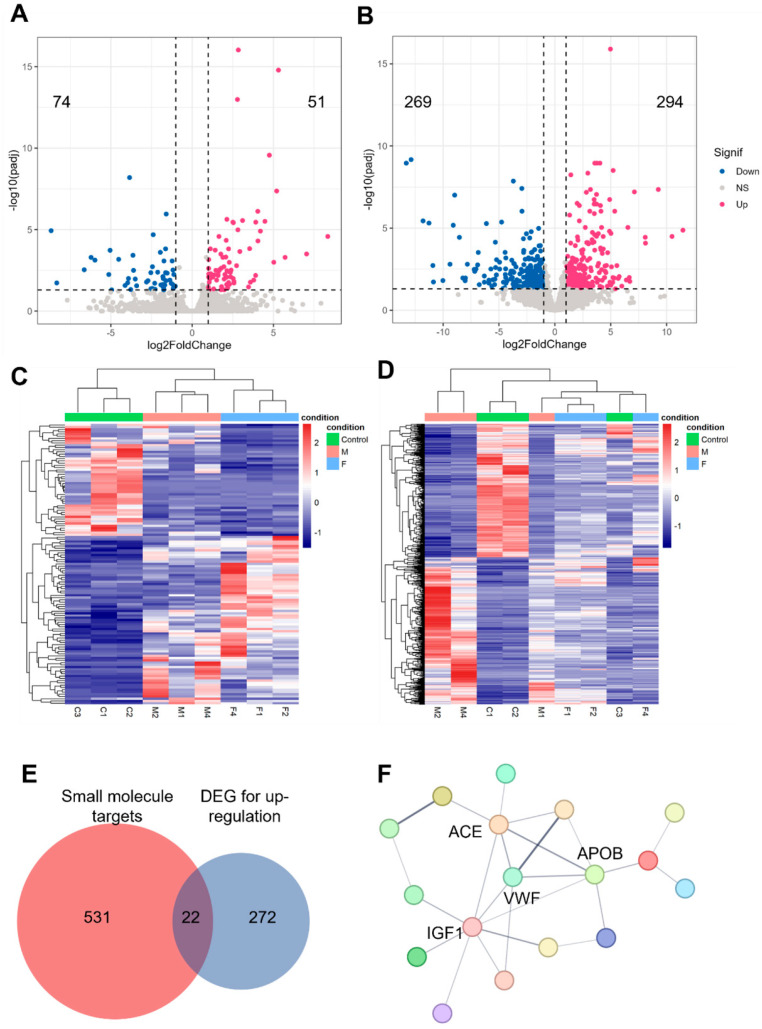
Identification of candidate targets potentially associated with AED-mediated disease modulation. (**A**,**B**) Volcano plots showing transcriptomic alterations following DSS-induced injury and subsequent AED treatment. DEGs were defined as those with |log_2_(fold change)| ≥ 1.0 and adjusted *p* < 0.05. (**C**,**D**) Heatmaps depicting DEGs corresponding to the datasets in panels (**A**) and (**B**). (**E**) Venn diagram illustrating the overlap between AED-upregulated DEGs and predicted small-molecule targets. (**F**) Protein–protein interaction network constructed from the 22 overlapping DEGs.

To gain insight into the molecular pathways associated with AED intervention in DSS-induced colitis, colon tissues from the control, DSS, and HAED groups were subjected to transcriptomic analysis to identify DEGs. As shown in Figure 5A,C, DSS exposure was associated with marked transcriptomic changes relative to controls, yielding 125 DEGs (51 upregulated and 74 downregulated). GO enrichment of the DSS-upregulated genes (Supplementary Figure S3) showed over-representation of inflammation-related biological processes, including Toll-like receptor 4 (TLR4) binding, consistent with previous reports implicating TLR4-linked inflammatory responses in DSS colitis [37,38,39].

Relative to controls, HAED treatment was associated with 563 DEGs (294 upregulated and 269 downregulated; Figure 5B,D). Functional enrichment analyses (Supplementary Figures S4 and S5) indicated that HAED-related DEGs were enriched in inflammation-associated and extracellular matrix (ECM)-related terms, including SLC-mediated transmembrane transport and regulation of TLR signaling by endogenous ligands. Metascape enrichment further highlighted the NABA matrisome category among HAED-associated gene sets, pointing to ECM-related transcriptomic remodeling. In line with this pattern, transcripts linked to matrix remodeling—including collagen cross-linking enzymes (LOX, P4HA1) and matrix metalloproteinases (MMP2, MMP9)—were increased in the HAED group.

To explore candidate mediators of HAED, transcriptomic profiles were integrated with small-molecule interaction information derived from UPLC–MS/MS profiling and literature curation. This integration yielded 553 putative HAED-interacting genes, of which 22 overlapped with HAED-upregulated DEGs (Figure 5E). Protein–protein interaction analysis of the overlapping genes identified ACE, APOB, IGF1, and VWF as highly connected nodes (Figure 5F and Supplementary Table S3). These molecules were considered candidate targets for further exploratory analysis rather than definitively validated mediators. ACE and VWF were subsequently selected for exploratory computational assessment, with representative molecular dynamics analysis further conducted for ACE (Supplementary Table S4).

### 3.5. Exploratory Computational Analysis of Representative AED Compounds and Candidate Targets

**Figure 6:**
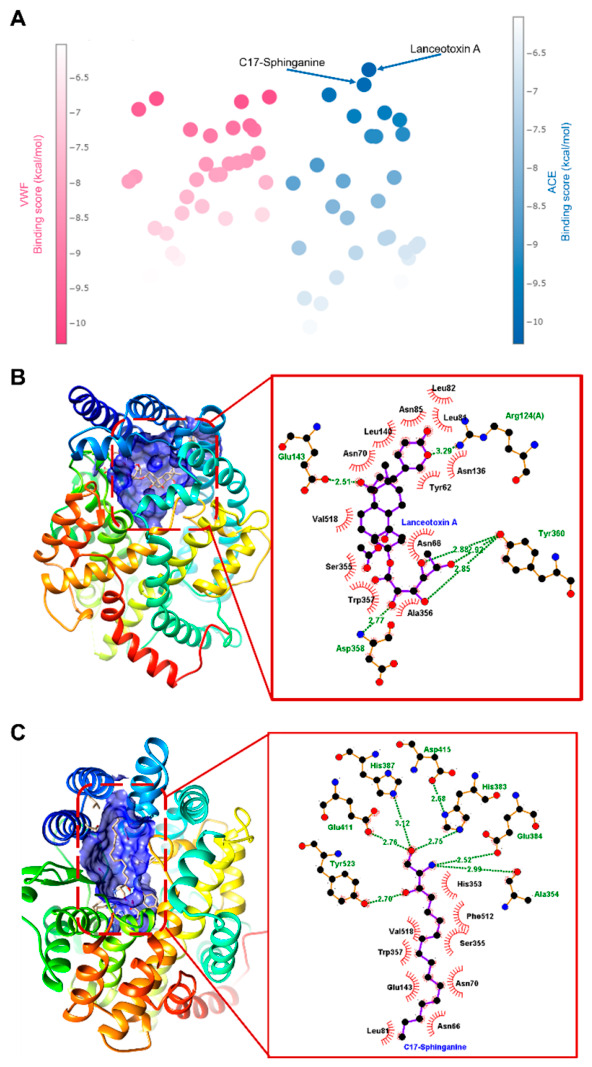
Exploratory molecular docking analysis of representative AED compounds with candidate targets. (**A**) Predicted binding affinities (kcal/mol) of representative HAED compounds with the candidate proteins ACE and VWF. (**B**,**C**) Representative predicted docking models of ACE with Lancerotoxin A and C17-Sphinganine.

To explore potential interactions between representative AED compounds and candidate protein targets relevant to colitis, we performed an exploratory computational analysis of candidate targets identified from integrative analysis, with detailed structural characterization focused on ACE. As shown in Figure 6A, several representative compounds displayed relatively favorable predicted binding scores with these candidate proteins, including docking scores below −10 kcal/mol for some ligand–target pairs (Supplementary Table S5). Among them, Lancerotoxin A and C17-Sphinganine were selected as representative compounds for visualization of their predicted docking poses with ACE (Figure 6B,C). Both compounds showed predicted binding compatibility with ACE, and MM/PBSA analysis based on molecular dynamics simulations supported relatively favorable predicted binding free energies in this computational setting (Supplementary Tables S6–S8).

**Figure 7:**
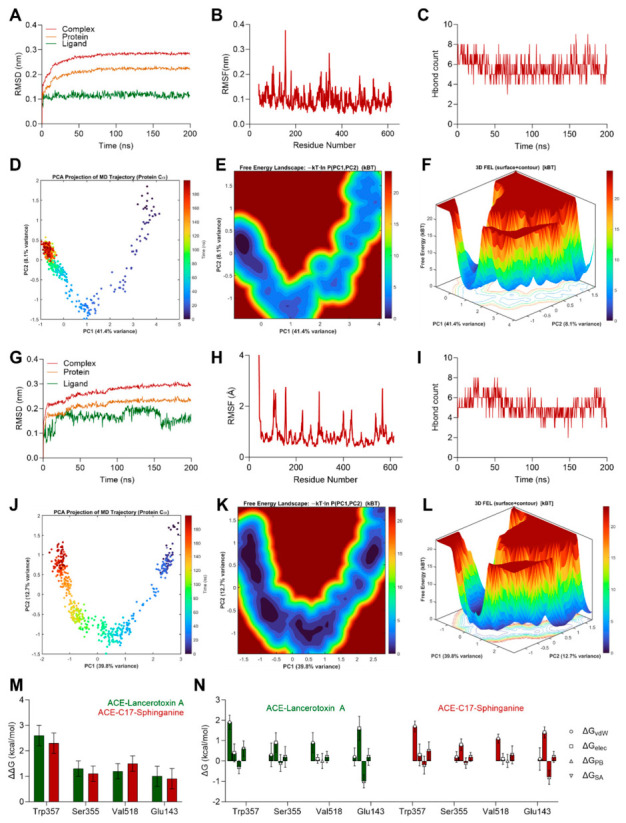
Molecular dynamics and energetic analyses of ACE–ligand interactions. (**A**–**F**) ACE–Lancerotoxin A complex: RMSD of the complex, protein and ligand (**A**); per-residue RMSF (**B**); time course of protein–ligand hydrogen bonds (**C**); PCA projection of dominant motions (**D**); two-dimensional free-energy landscape (2D–FEL) (**E**); and three-dimensional FEL representation (**F**). (**G**–**L**) ACE-C17–Sphinganine complex: corresponding RMSD (**G**), RMSF (**H**), hydrogen bonds (**I**), PCA projection (**J**), 2D–FEL (**K**), and 3D–FEL (**L**). (**M**) Predicted binding free-energy changes (ΔΔG) from MM/PBSA alanine scanning of conserved hot-spot residues (Trp357, Ser355, Val518, and Glu143) shared by both complexes. (**N**) Energy-component decomposition (ΔE_vdW, ΔE_elec, ΔG_PB, and ΔG_SA) for the alanine mutants. Data are mean ± SD.

To further characterize these predicted interactions, molecular dynamics simulations were performed for the two representative ACE–ligand complexes. Overall, both complexes remained associated with ACE during the simulation period, although they exhibited distinct dynamic behaviors (Figure 7A–L). Compared with C17-Sphinganine, the ACE–Lancerotoxin A complex showed a relatively more stable conformational profile, with lower structural fluctuation and more persistent hydrogen-bonding interactions. In addition, MM/PBSA-based alanine scanning identified several shared candidate hot-spot residues, including Trp357, Ser355, Val518, and Glu143, that were predicted to contribute to ligand binding in both complexes (Figure 7M,N). Among them, Trp357 showed the largest predicted destabilizing effect upon alanine substitution.

Taken together, these results provide exploratory computational evidence for potential interactions between certain AED constituents and candidate targets identified from integrative transcriptomic and network analysis. Although ACE was selected for more detailed structural characterization as a representative example, both ACE and VWF remain preliminary candidate targets that require further experimental and functional validation.

### 3.6. Metabolomic Analysis of Effects of the AED Intervention in DSS-Induced Colitis Mice

**Figure 8:**
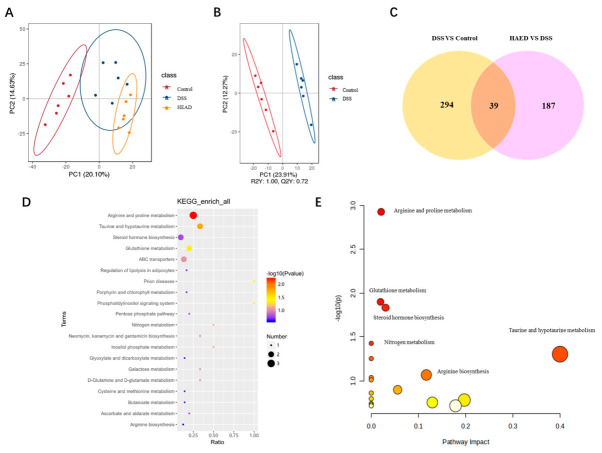
Metabolomic effects of AED on colonic tissues in DSS-induced ulcerative colitis mice. Untargeted LC–MS metabolomic profiling of colon samples (n = 6). (**A**) PCA score plot showing sample clustering. (**B**) PLS-DA score plot distinguishing DSS and control metabolic profiles. (**C**) Venn diagram of differential metabolites among groups. (**D**) KEGG pathway enrichment analysis based on significantly altered metabolites. (**E**) Summary of pathway impact analysis highlighting key metabolic pathways associated with AED treatment.

KEGG analysis of the transcriptome suggested that metabolic processes are associated with AED treatment; therefore, untargeted LC–MS metabolomics was performed to profile metabolic alterations in DSS-induced colitis. PCA showed clear separation between the DSS and Control groups, with the AED group located in an intermediate position (Figure 8A). PLS-DA further discriminated DSS from Control (Figure 8B). In total, 39 metabolites met the screening criteria (VIP > 1 and *p* < 0.05), and 36 displayed a “low–high–low” or “high–low–high” trend across Control–DSS–AED (Figure 8C; Supplementary Table S9). KEGG enrichment highlighted taurine-hypotaurine, arginine-proline, steroid hormone biosynthesis, glutathione, and nitrogen metabolism as principal AED-responsive pathways (Figure 8D,E).

At the metabolite level, AED was associated with coordinated shifts in redox-related metabolites, membrane lipids, eicosanoid derivatives, and steroid hormones. Hypotaurine decreased in DSS and increased after AED, and spermine elevation in DSS was attenuated in the AED group, supporting the involvement of taurine/hypotaurine metabolism and arginine/polyamine-related metabolism. Ether glycerophospholipids/plasmalogens (PC O-18:3/20:3/20:4/20:5), LPI/LPC species, and phytosphingosine reflected DSS-associated membrane remodeling that was directionally corrected by AED, aligning with the restoration of tight-junction proteins. Eicosanoid-related metabolites shifted with increased DiHETs and decreased 14,15-EET-glycerol, while corticosterone and tetrahydrocortisone increased with DSS and decreased after AED. Several central carbon/organic-acidrelated metabolites (2-hydroxycaproic acid, 2-hydroxyvaleric acid, glutamate, ribose, and myo-inositol) also showed partial normalization.

Overall, these data indicate that AED is associated with alterations in multiple metabolic pathways in DSS colitis, with shared transcriptome-metabolome enrichment in arginine biosynthesis, amino acid biosynthesis, and fat digestion and absorption, thereby providing candidate pathways and biomarkers for future validation.

### 3.7. Integrative Multi-Omics Analysis Identifies Key Molecular Drivers and Coordinated Reprogramming

A bipartite correlation network was constructed to characterize associations between the colonic transcriptome and metabolome within the DSS group (Supplementary Figure S6). The network comprised 48 nodes and 63 edges, with positive correlations accounting for 65% of connections. Based on composite centrality (H = C_D + C_B), L-hydroxyproline and dehydroepiandrosterone (DHEA) were identified as major metabolic hubs, while Creb3l3 was the most central gene node.

To further capture higher-order patterns beyond pairwise associations, singular value decomposition (SVD) was applied to the integrated multi-omics matrix (Supplementary Figure S7). Factor 2, explaining 48.7% of the total variance, represented a major axis of coordinated variation linking epithelial and metabolic features. Notably, Slc9a3 (NHE3) and Retnlb exhibited the highest loading values and co-varied with long-chain polyunsaturated fatty acids (PUFAs), including docosahexaenoic acid (DHA). This pattern suggests coordinated variation between epithelial ion transport, antimicrobial response, and lipid metabolism.

## 4. Discussion

Current strategies for UC management primarily aim to control inflammation, maintain remission, prevent complications, and improve quality of life [40]. However, conventional pharmacotherapy has significant limitations, including incomplete responses and the risk of cumulative adverse effects during long-term use [41]. Given these challenges, increasing attention has been directed toward dietary interventions and food-derived bioactive components as complementary approaches for intestinal health management [20]. Multiple classes of food-origin bioactive constituents (e.g., polysaccharides, polyphenols, and saponins) have been reported to ameliorate UC-relevant phenotypes by modulating inflammatory responses [21,42,43], oxidative stress [22], epithelial barrier integrity [44], and the gut microbiota-metabolism axis [40]. Previous studies, such as those examining green pea hull polyphenols [15] and cherry polyphenols [16], have demonstrated the potential of plant-derived bioactives to mitigate inflammation and improve gut health in UC. Likewise, DJF has been used as a raw material to obtain bioactive extracts or fractions with anti-inflammatory and antioxidant activities, supporting its potential as a source of functional ingredients [24,26]. However, while these studies primarily focus on inflammatory modulation, our study shows that AED not only reduces inflammation but also restores epithelial barrier integrity, highlighting its dual action in UC management. These findings suggest that AED may have potential as a food-derived functional ingredient for further preclinical and translational evaluation.

Inflammation is a coordinated host response to harmful stimuli, and pro-inflammatory mediators such as TNF-α, IL-6, and IL-1β contribute to UC pathogenesis by promoting immune-cell activation and infiltration, leading to mucosal injury and impaired repair [45]. Therefore, limiting excessive inflammatory activation and preventing its persistence are key objectives in UC intervention. Using an LPS-stimulated RAW264.7 macrophage model, we observed that AED reduced LPS-induced expression of inflammatory cytokines and inflammation-associated proteins, including iNOS and COX-2 [46], supporting its role as a dietary bioactive capable of modulating inflammatory responses at the cellular level. Notably, AED decreased the phosphorylation levels of JAK1 and STAT3, suggesting that modulation of JAK1/STAT3 signaling may be involved in its anti-inflammatory effects. JAK-STAT pathway activation is implicated in UC, and AED’s modulation of this pathway suggests its potential anti-inflammatory effects [35,47]. However, because no pathway inhibition or rescue experiments were performed in the present study, the contribution of JAK1/STAT3 should be interpreted as associative rather than causally established. Together, these findings support the involvement of JAK1/STAT3 signaling as a plausible intracellular route through which AED may support gut immune balance and barrier function.

Building on the in vitro findings, we further assessed AED in a DSS-induced colitis model, which recapitulates major clinical and histopathological features of UC and is widely used to investigate inflammatory mechanisms and evaluate anti-colitis interventions [48]. AED intervention alleviated colitis-associated manifestations, in parallel with SASP. In UC, inflammatory mediators can directly impair epithelial structure and function [45], and activated immune cells amplify these effects by producing additional cytokines that further compromise barrier integrity [49]. Disruption of tight junction proteins (e.g., claudin-1 and occludin) increases intestinal permeability, facilitating the translocation of luminal factors and exacerbating mucosal inflammation. Barrier disruption can also promote microbiota dysbiosis, which further fuels inflammatory responses [50], thereby reinforcing a self-sustaining inflammatory loop [51]. In this study, AED reduced the mRNA expression of pro-inflammatory mediators and increased the expression of key mucosal barrier proteins, including ZO-1, occludin, and claudin-1, supporting the possibility that AED may alleviate colitis in association with reduced inflammatory responses and improved epithelial barrier homeostasis.

Network pharmacology analysis identified ACE and VWF as potential targets of AED, which will be further investigated in future studies. Earlier work reported reduced ACE activity in active Crohn’s disease and UC, potentially reflecting mucosal injury at intestinal production sites, together with elevated activities of carboxypeptidase N1 and N2 (CPN1, CPN2), suggesting dysregulated kininase activity in inflammatory bowel disease [52]. Such protease-related dysregulation may further influence epithelial homeostasis, including pathways linked to barrier function. Consistently, angiotensin II has been reported to suppress intestinal P-glycoprotein function and surface expression via AT1R-dependent PI3K-Akt-p38 MAPK signaling, contributing to epithelial dysfunction during inflammation [53]. For the VWF axis, ADAMTS13 deficiency has been shown to exacerbate colitis by promoting VWF-mediated platelet–leukocyte recruitment and intestinal thrombosis, whereas recombinant ADAMTS13 ameliorates disease severity, highlighting VWF/ADAMTS13 as a potential therapeutic axis in inflammatory bowel disease. Moreover, ER stress-related genes, including VWF, have been implicated in immune-mediated intestinal fibrosis in UC, and IKK-16 has been proposed as a candidate targeting this pathogenic axis [54]. Based on these lines of evidence, ACE and VWF were selected for subsequent molecular docking, molecular dynamics simulations, and related analyses to explore potential AED–target interactions. These computational analyses are exploratory and serve mainly to generate hypotheses for future target-validation studies.

Omics approaches provide a systems-level perspective that can help interpret the holistic and dynamic actions of multi-component natural products [55]. Here, transcriptomics and metabolomics were integrated to delineate further pathways associated with AED intervention. Transcriptomic analysis highlighted pathways related to lipid metabolism and microbial–host interactions, which may contribute to AED’s effects in UC. Transporters represent critical components of mucosal barrier function and can influence both disease progression and therapeutic responses. In inflamed UC tissues, reduced expression of metabolic enzymes (e.g., CYP2C9, UGT1A1) and transporters (e.g., ABCB1, ABCG2) has been reported, while specific uptake transporters may be increased [56]. GO analysis also supported a link between IBD pathogenesis and lipid transport. Microbiota-derived short-chain fatty acids, often reduced in IBD mucosa, are essential for intestinal homeostasis and can signal through GPCRs (e.g., GPR41, GPR43, and GPR109A) to modulate immune function [57,58]. In addition, KEGG enrichment analysis highlighted bile acid secretion, consistent with evidence that bile acid recycling and ileal/colonic absorption are frequently impaired in IBD due to downregulation of bile acid transporters, potentially contributing to disease persistence and progression [59].

Metabolomics revealed AED-associated changes in metabolites and pathways relevant to UC pathophysiology. Taurine-hypotaurine metabolism emerged as a prominent pathway, consistent with taurine’s known roles in antioxidant defense and anti-inflammatory regulation. Arginine-linked pathways, including arginine-creatine and arginine–polyamine metabolism, were also implicated; previous studies reported that arginase-1 (Arg1) and the creatine transporter Slc6a8 can attenuate inflammatory activation in macrophages by reducing iNOS expression and pro-inflammatory cytokine production [60]. Collectively, the integrated transcriptome–metabolome results support the view that AED is associated with coordinated changes in inflammatory, transport-related, and metabolic pathways. However, these data do not by themselves establish a causal metabolism–JAK1/STAT3–barrier axis, which will require direct functional validation in future studies.

## 5. Conclusions

AED showed protective effects in experimental models of ulcerative colitis by attenuating intestinal inflammation, reducing NO production and pro-inflammatory cytokines (IL-6, IL-1β, and TNF-α) without detectable cytotoxicity, and was associated with suppression of JAK1/STAT3 activation. In DSS-induced colitis, AED improved disease activity and histopathological features, accompanied by reduced inflammatory infiltration and increased expression of epithelial barrier proteins. Integrated transcriptomic and metabolomic analyses further indicated that AED intervention was associated with coordinated modulation of candidate targets and multiple metabolic pathways relevant to UC pathophysiology. Collectively, these findings support AED as a standardized food-derived functional ingredient with potential application in dietary management of intestinal inflammation and gut health maintenance.

Given that this study is preclinical, future research should focus on validating AED’s effects in more clinically relevant models, such as humanized animal models or intestinal organoid cultures, which more accurately replicate the human intestinal microenvironment. These models will provide a better understanding of AED’s efficacy and safety in human UC treatment. In particular, pathway inhibition or rescue experiments will be necessary to determine whether the candidate signaling and metabolic pathways identified here are causally involved in AED-mediated protection. Further studies evaluating long-term use and safety will also help to support the future development of AED. While AED has shown promising effects in modulating inflammation and enhancing intestinal barrier function, further exploration of its potential as a functional food ingredient for UC management is needed. Long-term studies in dietary interventions should also be conducted, considering both their efficacy and safety as a non-pharmacological therapeutic approach to UC.

## Data Availability

The original contributions presented in this study are included in the article/Supplementary Materials. Further inquiries can be directed to the corresponding authors.

## Supplementary Materials

The following supporting information can be downloaded at: https://www.mdpi.com/article/10.3390/foods15111866/s1, Figure S1: UPLC-QTOF-MS/MS data of the AED extract obtainedtitle; Figure S2: Effects of AED on cell viability and nitric oxide (NO) secretion in RAW264.7 macrophages; Figure S3: Functional and pathway enrichment analysis of upregulated DEGs for DSS-induced disease model; Figure S4: Functional and pathway enrichment analysis of upregulated DEGs; Figure S5: Pathway enrichment analysis of therapy-associated up-regulated DEGs identified by Metascape; Figure S6: Integrated metabolite–gene association network in colitis. Figure S7: Singular value decomposition identifies dominant axes of integrated multi-omics variation; Table S1: Real-Time PCR assay Primer sequence; Table S2: AED molecular marker to pathway; Table S3: Biological function of identified critical gene and relative function; Table S4: Structure information of related protein; Table S5: Binding affinities of selected small molecules toward ACE and VWF; Table S6: MM/PBSA calculations for these two complexes; Table S7: Per-residue free-energy decomposition of Lancerotoxin A-ACE; Table S8: Per-residue free-energy decomposition of C17-Sphinganine-ACE; Table S9: Differential metabolites in the colon of each group;.
