# Supplementary material for: Walnut Septum-Derived Aqueous Extract Alleviates Colitis Through Modulation of Gut Metabolism and Inflammatory Signaling
Source: Foods. 2026 May 25;15(11):1866. doi: 10.3390/foods15111866 (PMC13256698; doi:10.3390/foods15111866)
Supplement: Supplementary file 1 [file foods-15-01866-s001.zip › foods-4220650-supplementary.pdf]

foods

MDPI

Article

Walnut septum-derived aqueous extract alleviates colitis through modulation of gut metabolism and inflammatory signaling

Beier Jiang[1,†], Yu Wan[1,†] Lina Liu[1], Jiajun Cheng[2], Tianjiao Min[1], Xinlong Gao[1], Zicheng Yu[2], Li Ma[1,*], Ying He[1,*]

[1] Navy Medical Centre, Naval Medical University, Shanghai 200433, China
[2] College of Food Science and Technology, Shanghai Ocean University, Shanghai 201306, China
* Correspondence: marycnn@163.com(L.M.); liulina0106@smmu.edu.cn (L.L.); yinghe@smmu.edu.cn (Y.H.)
† These authors contributed equally to this work.

Table S1. Real-Time PCR assay Primer sequence

Gene		Primer sequences(5'-3')
Mouse-GADPH	Forward	ACTCCACGACATACTCAGC
Mouse-GADPH	Reverse	TCAACGGCACAGTCAAGG
Mouse-iNOS	Forward	ACCCAAGGTCTACGTTCAGG
Mouse-iNOS	Reverse	CGCACATCTCCGCAAATGTA
Mouse-IL-6	Forward	TGGGACTGATGCTGGTGACAAC
Mouse-IL-6	Reverse	AGCCTCCGACTTGTGAAGTGGT
Mouse-IL-1β	Forward	GAAATGCCACCTTTTGACAGTG
Mouse-IL-1β	Reverse	TGGATGCTCTCATCAGGACAG
Mouse-TNF-α	Forward	CCCTCACACTCAGATCATCTTCT
Mouse-TNF-α	Reverse	GCTACGACGTGGGCTACAG

Academic Editor: Alberto Cepeda Sáez

Received: 10 March 2026
Revised: 30 April 2026
Accepted: 18 May 2026
Published: 25 May 2026

Copyright: © 2026 by the authors. Licensee MDPI, Basel, Switzerland. This article is an open access article distributed under the terms and conditions of the Creative Commons Attribution (CC BY) license.

Table S2. AED molecular marker to pathway.

Analyte	Mode	RT_min	Q1_mz	Putative_pathways
Quercitrin	Positive	15.1175	448.0998	Flavonoid/Phenylpropanoid metabolism; Antioxidant/Nrf2 signaling; Barrier/TJ support
C17-Sphinganine	Positive	23.26017	287.2819	Sphingolipid metabolism; Membrane/barrier signaling; Inflammatory modulation
6-Methoxyflavonol	Negative	0.829333	268.0699	Flavonoid/Phenylpropanoid metabolism; Antioxidant/Nrf2 signaling; Barrier/TJ support
Succinic acid	Negative	1.371	118.0228	TCA cycle; Mitochondrial energetics; Inflammation Metabolism coupling
Catechin	Negative	8.433333	290.0679	Flavonoid/Phenylpropanoid metabolism; Antioxidant/Nrf2 signaling; Barrier/TJ support

Table S3. Biological function of identified critical gene and relative function.

Gene	Biological function
ACE	angiotensin I converting enzyme. It catalyzes the conversion of angiotensin I into a physiologically active peptide angiotensin II. Angiotensin II is a potent vasopressor and aldosterone-stimulating peptide that controls blood pressure and fluid-electrolyte balance. This angiotensin converting enzyme (ACE) also inactivates the vasodilator protein, bradykinin.
APOB	apolipoprotein B. This gene product is the main apolipoprotein of chylomicrons and low density lipoproteins (LDL), and is the ligand for the LDL receptor. It occurs in plasma as two main isoforms, apoB-48 and apoB-100: the former is synthesized exclusively in the gut and the latter in the liver.
IGF1	insulin like growth factor 1. The protein encoded by this gene is similar to insulin in function and structure and is a member of a family of proteins involved in mediating growth and development.
VWF	von Willebrand factor. This gene encodes a glycoprotein involved in hemostasis. The encoded preproprotein is proteolytically processed following assembly into large multimeric complexes. These complexes function in the adhesion of platelets to sites of vascular injury and the transport of various proteins in the blood.

Table S4. Structure information of related protein.

Gene	PDB ID	Resolution	Ligand
ACE	3L3N	2.30 Å	N~2~-[(1S)-1-carboxy-3-phenylpropyl]-L-lysyl-L-tryptophan
VWF	3HXQ	2.69 Å	DNA Aptamer ARC1172

https://doi.org/10.3390/foods15111866

Table S5. Binding affinities of selected small molecules toward ACE and VWF.

Compound_name	ACE_Score_kcal_per_mol	ACE_Affinity	VWF_Score_kcal_per_mol	VWF_Affinity
Homocysteine thiolactone	-8.54	Moderate	-8.17	Moderate
2-benzylsulfanyl-N-[[1-(hydroxymethyl)cyclopropyl]methyl]acetamide	-7.53	Moderate	-8.25	Moderate
Muscomosin	-6.69	Weak	-9.61	Strong
2-(4-Methoxyphenyl)naphthalic anhydride	-6.17	Weak	-6.08	Weak
N-[(4-ethoxyphenyl)methyl]-5,6,7,8-tetrahydroimidazo[1,2-a]pyridine-2-carboxamide	-8.76	Moderate	-7.1	Strong
Combrestatin A4	-6.47	Weak	-9.83	Strong
Quercitrin	-9.84	Strong	-6.93	Weak
Hexahydrocurcumin	-8.04	Moderate	-6.45	Weak
(5S,8R)-8-(but-3-en-1-yl)-5-((E)-pent-2-en-4-yn-1-yl)octahydroindolizine	-6.8	Weak	-6.29	Weak
CAY10590	-8.43	Moderate	-7.31	Weak
Prostaglandin I2	-7.78	Moderate	-7.66	Moderate
Fluorescein	-9.5	Strong	-9.16	Strong
Dihydrogranaticin	-7.1	Weak	-8.02	Moderate
C17-Sphinganine	-10.03	Strong	-7.38	Weak
Lanceotoxin A	-10.29	Strong	-8.35	Moderate
Met His Lys	-9.14	Strong	-9	Moderate
SPB 22:0;2O	-9.13	Strong	-8.07	Moderate
6-Methoxyflavonol	-7.93	Moderate	-8.07	Moderate
Met-Ala-OH	-9.55	Strong	-6.97	Weak
3-O-methylfunicone	-7.16	Weak	-8.3	Moderate
9-Phenyl-6-sulfanylidene-3H-purin-2-one	-7.16	Weak	-9.16	Moderate
Dehydrorotenone	-8.31	Moderate	-8.3	Moderate
Succinic acid	-9.41	Strong	-9.79	Strong
Diphenylphosphine Acid	-9.13	Strong	-8.33	Moderate
DIPLOSALSALATE	-6.11	Weak	-9.2	Strong
Lactobionic acid	-6.7	Weak	-9.09	Strong
[3,4,5-trihydroxy-6-(3,4,5-trihydroxybenzoyl)oxyoxan-2-yl]methyl 3,4,5-trihydroxybenzoate	-6.54	Weak	-8.49	Moderate
Catechin	-6.03	Weak	-6.86	Weak
2-Glucosyloxy-4-methoxycinnamic acid	-6.72	Weak	-7.7	Moderate
Ellagic acid	-6.76	Weak	-9.92	Strong

Note: Binding affinities were classified as strong (≤ −9.0 kcal·mol^{-1}), moderate (−9.0 to −7.5 kcal·mol^{-1}), or weak (> −7.5 kcal·mol^{-1}) based on AutoDock-style binding energy, with more negative values indicating stronger ligand–protein interactions. AutoDock-style binding energies are expressed in kcal·mol^{-1}. More negative values denote stronger binding affinity. The thresholds (−9.0 and −7.5 kcal·mol^{-1}) were chosen according to commonly reported docking benchmarks for small-molecule–protein interactions, where values below −9 kcal·mol^{-1} typically indicate strong and specific binding.

Table S6. MM/PBSA calculations for these two complexes.

Lancerotoxin A-ACE		C17-Sphinganine	
Index	Mean ± SD	Index	Mean ± SD
ΔEvdW	−48.7 ± 2.3	ΔEvdW	−36.5 ± 2.1
ΔEele	−17.9 ± 3.1	ΔEele	−22.1 ± 3.0
ΔGpolar (PB)	+30.6 ± 2.8	ΔGpolar (PB)	+30.9 ± 2.7
ΔGnonpolar (SA)	−6.2 ± 0.5	ΔGnonpolar (SA)	−5.4 ± 0.4
ΔG_bind (no −TΔS)	−42.2 ± 2.7	ΔG_bind (no −TΔS)	−33.1 ± 2.8

Table S7. Per-residue free-energy decomposition of Lancerotoxin A-ACE.

Residue	ΔG_res (tot)	vdW	Elec	PB	SA	Notes
Leu82	−3.9	−4.5	−0.2	+1.1	−0.3	Hydrophobic pocket anchor
Leu84	−3.5	−4.0	−0.1	+0.8	−0.2	Hydrophobic packing with core
Tyr62	−3.1	−2.6	−1.2	+0.9	−0.2	π/edge + H-bond donor/acceptor
Asn68	−2.7	−1.4	−2.3	+1.2	−0.2	H-bond (ligand carbonyl)
Asn70	−2.3	−1.1	−2.0	+1.1	−0.3	H-bond network
Arg124	−2.9	−0.8	−5.1	+3.3	−0.3	Salt-bridge/H-bond to heteroatom
Asn136	−1.6	−0.9	−1.4	+0.9	−0.2	Polar contact
Leu140	−1.8	−2.1	−0.1	+0.5	−0.1	Hydrophobic buttress
Glu143	−0.8	−0.4	−2.7	+2.5	−0.2	H-bond; mild desolvation penalty
Ser355	−1.2	−0.6	−1.3	+0.8	−0.1	H-bond (side-chain OG)
Ala356	−0.9	−1.0	0.0	+0.2	0.0	Backbone contact
Trp357	−2.4	−2.7	−0.3	+0.7	−0.1	π-stack/dispersion with ligand ring
Tyr360	−1.7	−1.8	−0.4	+0.6	−0.1	π-hydrophobic
Val518	−1.1	−1.3	0.0	+0.2	0.0	Hydrophobic contact

Table S8. Per-residue free-energy decomposition of C17-Sphinganine -ACE.

Residue	ΔG_res (tot)	vdW	Elec	PB	SA	Notes
Leu82	−3.9	−4.5	−0.2	+1.1	−0.3	Hydrophobic pocket anchor
Leu84	−3.5	−4.0	−0.1	+0.8	−0.2	Hydrophobic packing with core
Tyr62	−3.1	−2.6	−1.2	+0.9	−0.2	π/edge + H-bond donor/acceptor
Asn68	−2.7	−1.4	−2.3	+1.2	−0.2	H-bond (ligand carbonyl)
Asn70	−2.3	−1.1	−2.0	+1.1	−0.3	H-bond network
Arg124	−2.9	−0.8	−5.1	+3.3	−0.3	Salt-bridge/H-bond to heteroatom
Asn136	−1.6	−0.9	−1.4	+0.9	−0.2	Polar contact
Leu140	−1.8	−2.1	−0.1	+0.5	−0.1	Hydrophobic buttress
Glu143	−0.8	−0.4	−2.7	+2.5	−0.2	H-bond; mild desolvation penalty
Ser355	−1.2	−0.6	−1.3	+0.8	−0.1	H-bond (side-chain OG)
Ala356	−0.9	−1.0	0.0	+0.2	0.0	Backbone contact
Trp357	−2.4	−2.7	−0.3	+0.7	−0.1	π-stack/dispersion with ligand ring
Tyr360	−1.7	−1.8	−0.4	+0.6	−0.1	π-hydrophobic
Val518	−1.1	−1.3	0.0	+0.2	0.0	Hydrophobic contact

Table S9. Differential metabolites in the colon of each group.

metabolites	DSS vs. NC			HEAD vs. DSS		
	P	VIP	Trend	P	VIP	Trend
Tetrahydrocortisone	0.000174	2.14653	up	0.007036	1.34667	down
2-(14,15-Epoxyeicosatrienoyl) glycerol	0.023088	1.48321	down	0.001205	1.80867	up

https://doi.org/10.3390/foods15111866

7-{[(2E)-3,7-dimethylocta-2,6-dien-1-yl]oxy}-2H-chromen-2-one	0.024322	1.16063	down	0.005092	2.08120	up
2-Hydroxycaproic acid	0.000263	1.62772	up	0.004017	2.08336	down
LPI 22:6	0.014019	1.40184	down	0.004552	1.84951	up
FAHFA 18:0/20:2	0.003932	1.55694	up	0.001344	2.01134	down
Hypotaurine	0.000128	2.21239	down	0.000974	1.80087	up
Clinafloxacin	0.000480	2.16020	up	0.008392	1.49079	down
Lysopc 16:2 (2N Isomer)	0.008452	1.84465	up	0.018055	1.72311	down
Maltopentaose	0.002154	1.53837	up	0.034447	1.57255	down
Corticosterone	7.77E-06	2.07342	up	0.011031	1.59469	down
Phytosphingosine	0.049419	1.24113	up	0.024530	1.48619	down
Prostaglandin K2	0.019252	1.50316	up	0.033699	1.67967	down
PC O-16:1	0.000881	2.04606	down	0.001839	1.52624	up
Nor-9-carboxy-δ9-THC	0.004736	1.78412	up	0.001175	1.73414	down
PC O-20:4	0.011104	1.25145	down	0.000642	2.37012	up
PC O-20:3	0.004064	1.56479	down	0.001178	2.15548	up
L-Hydroxyproline	0.002052	1.97615	down	0.015690	1.57936	up
PC O-18:3	0.000190	1.86012	down	0.006158	1.79615	up
2-Hydroxyvaleric acid	0.000238	1.86220	up	0.031048	1.39699	down
cis-7-Hexadecenoic Acid	0.038369	1.48451	down	0.030467	1.44087	up
SPB 16:1;2O	0.025891	1.23266	up	0.026578	1.66982	down
LPC 14:0-SN1	0.048776	1.36021	down	0.021643	1.43488	up
L-Cystathionine	0.009311	1.70999	down	0.013843	1.59039	up
Lysops 22:6	0.011055	1.25718	down	0.007731	2.02374	up
(±)8(9)-DiHET	0.003217	1.68669	up	0.013079	1.57194	down
PC O-20:5	0.012245	1.54996	down	0.012712	1.91072	up
LPC 18:3-SN1	0.002622	1.11082	down	0.004011	2.24250	up
L-Glutamic acid	0.006593	1.63247	down	0.000901	2.02127	up
PC 20:4_20:4	0.018445	1.35267	up	0.049806	1.48843	down
LPC 18:3	0.000652	1.32664	down	0.000393	2.40558	up
Spermine	0.045928	1.18331	up	0.018295	1.69111	down
5α-Dihydrotestosterone	0.008784	1.68800	down	0.005716	1.68343	up
D-Ribose	0.046902	1.26238	up	0.031821	1.55439	down
Inositol	0.010588	1.73142	up	0.007607	1.36980	down
2-(1H-benzimidazol-2-yl)-N-[4-(benzyloxy)phenyl]benzamide	0.00210	1.90166	up	0.029058	1.21571	down

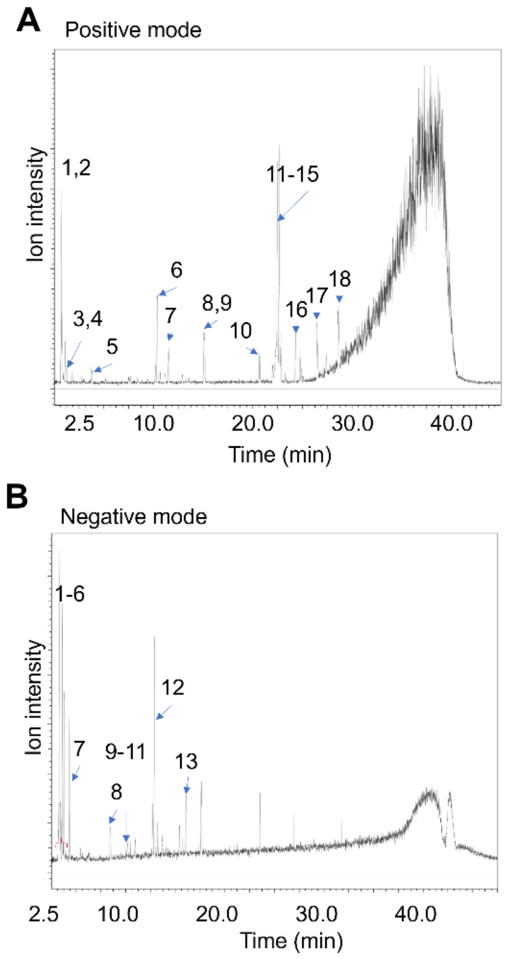

Figure S1. UPLC-QTOF-MS/MS data of the AED extract obtained. A. positive ion mode; B. negative ion mode.

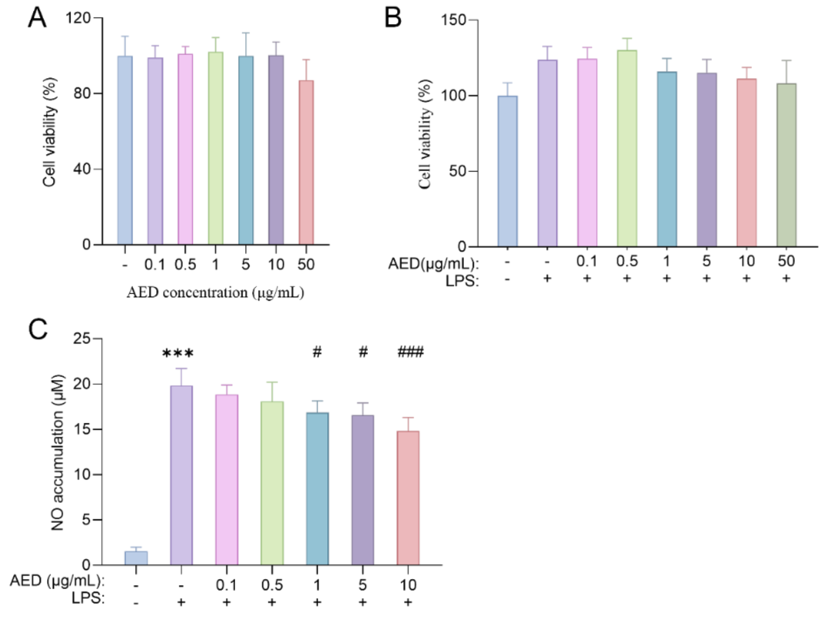

Figure S2. Effects of AED on cell viability and nitric oxide (NO) secretion in RAW264.7 macrophages. (A) Basal (unstimulated) condition. (B) LPS-stimulated condition. (C) Quantification of NO production following AED treatment. Data are presented as mean ± SD (n = 5). Statistical significance was assessed by one-way ANOVA followed by Dunnett's post-test. P values indicate

comparisons versus the Control group (*P < 0.05, **P < 0.01, ***P < 0.001), and # symbols denote comparisons versus the LPS group (#P < 0.05, ##P < 0.01, ###P < 0.001).

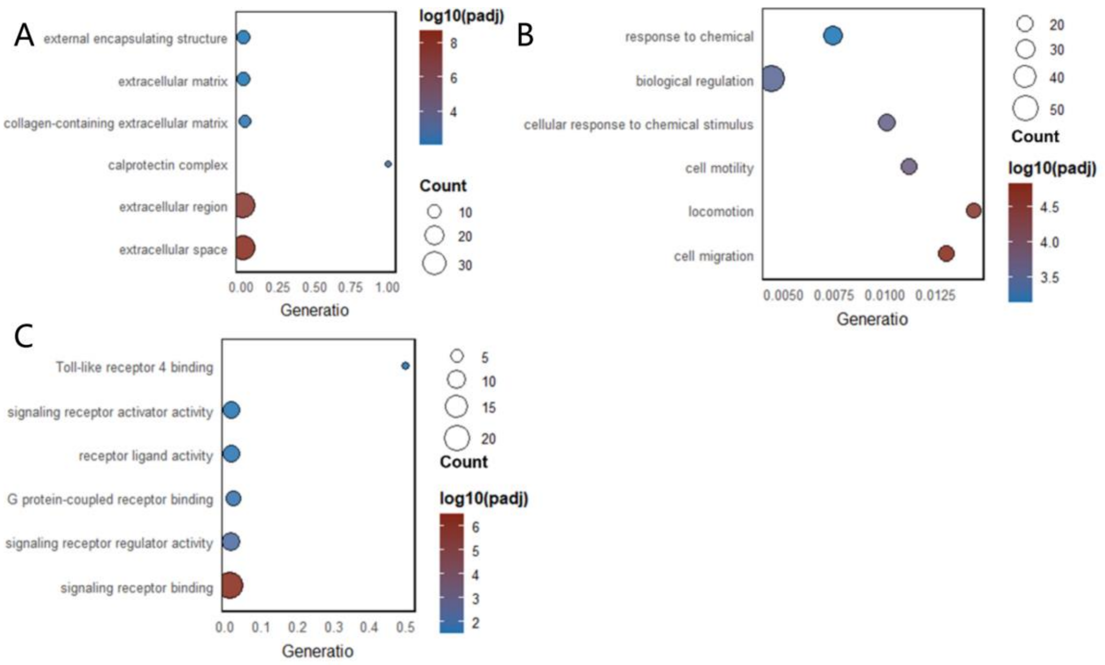

Figure S3. Functional and pathway enrichment analysis of upregulated DEGs for DSS-induced disease model. GO enrichment analysis summarizing significantly over-represented terms within the (A) molecular function. (B) biological process (BP). (C) cellular component (CC) categories.

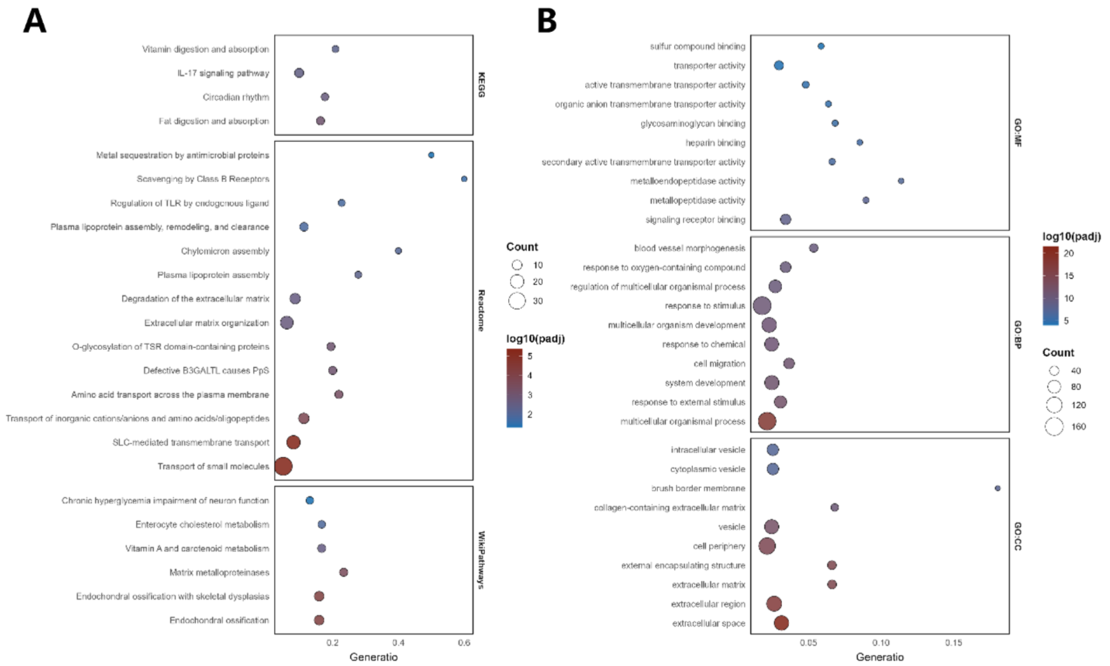

Figure S4. Functional and pathway enrichment analysis of upregulated DEGs. (A) GO enrichment analysis summarizing significantly over-represented terms within the molecular function, biological process, and cellular componentcategories. (B) Pathway enrichment analysis integrating results from the KEGG, Reactome, and WikiPathways databases. In both panels, the x-axis denotes the Generatio group, and the y-axis lists enriched terms or pathways. Bubble size corresponds to the number of annotated DEGs (Count), and bubble color represents the adjusted p-value on a log10 scale (log10 padj), ranging from blue to dark red.

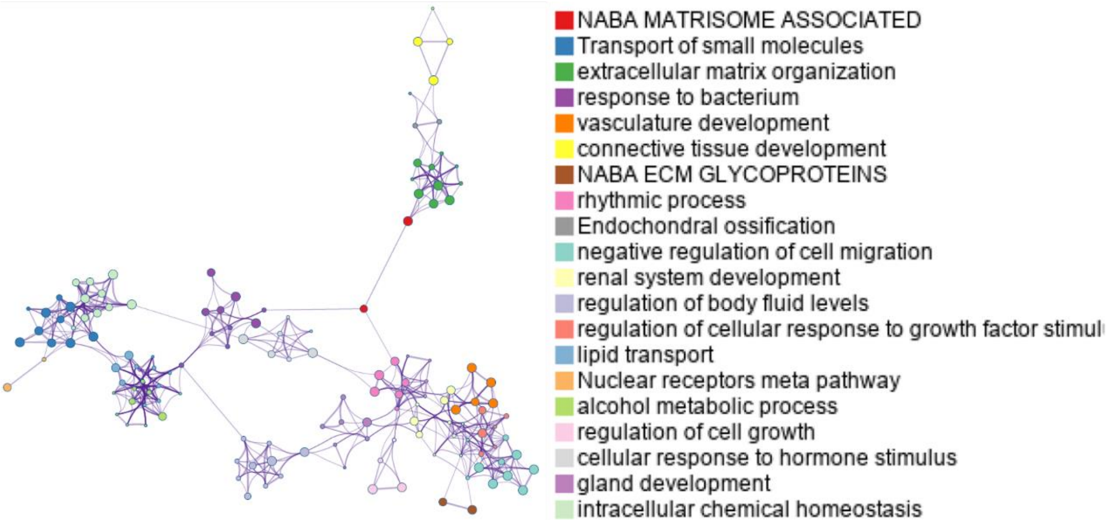

Figure S5. Pathway enrichment analysis of therapy-associated up-regulated DEGs identified by Metascape. Pathway enrichment analysis of 294 therapy-related DEGs performed using the Metascape platform.

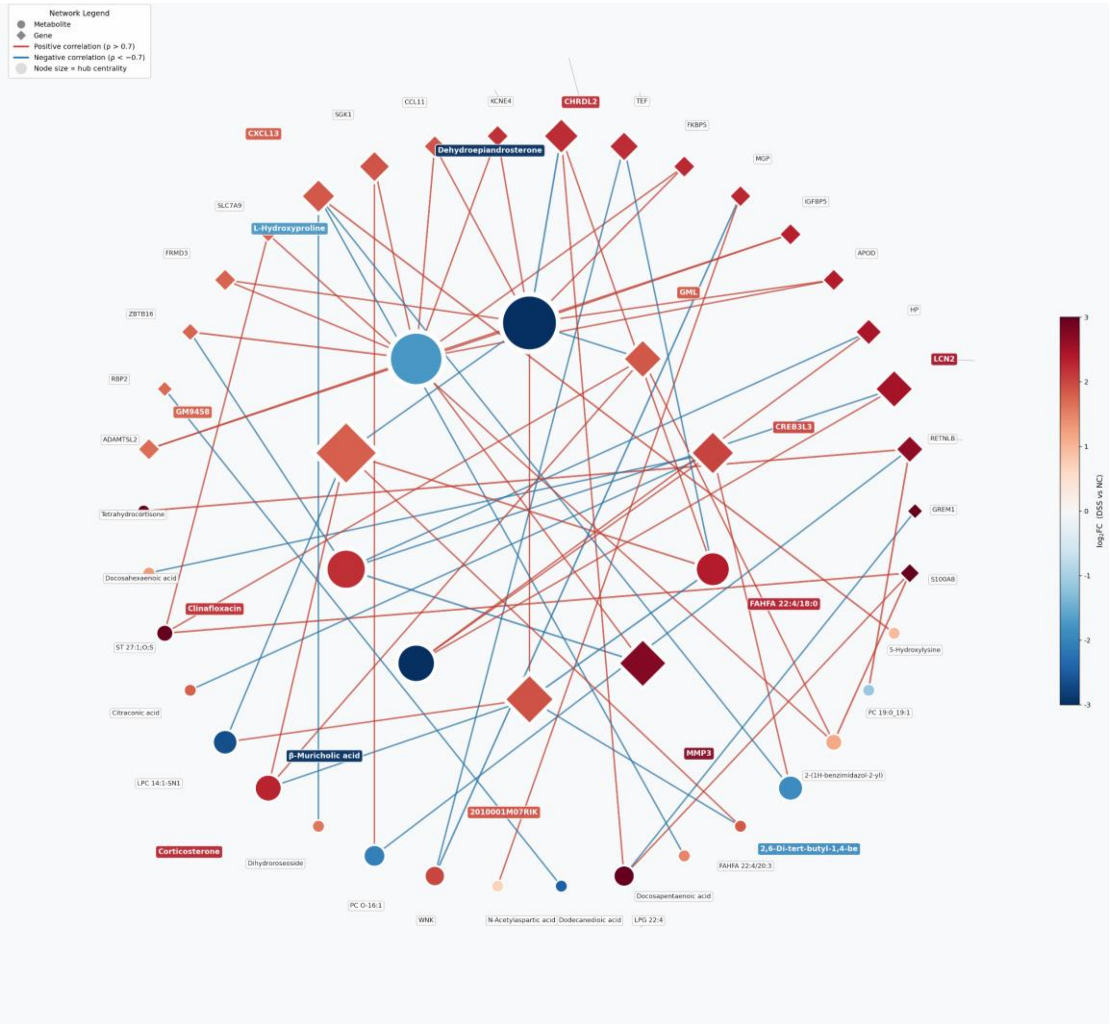

Figure S6. Integrated metabolite–gene association network in colitis .Bipartite network showing significant associations between colonic metabolites (circles) and transcripts (diamonds). Edges represent Spearman correlations with |ϱ|>0.7 and P<0.05. Edge color indicates correlation sign (red, positive; blue, negative), and edge width and opacity are scaled to |ϱ|. Node size reflects a composite centrality score derived from degree and betweenness centrality, and node color indicates

log_2(foldchange) (DSS versus Control). Labels are shown for top-tier hubs ranked in the top 30% of composite centrality. The network is displayed using a dual-ring shell layout. n=10 samples total (5 mice per group) .

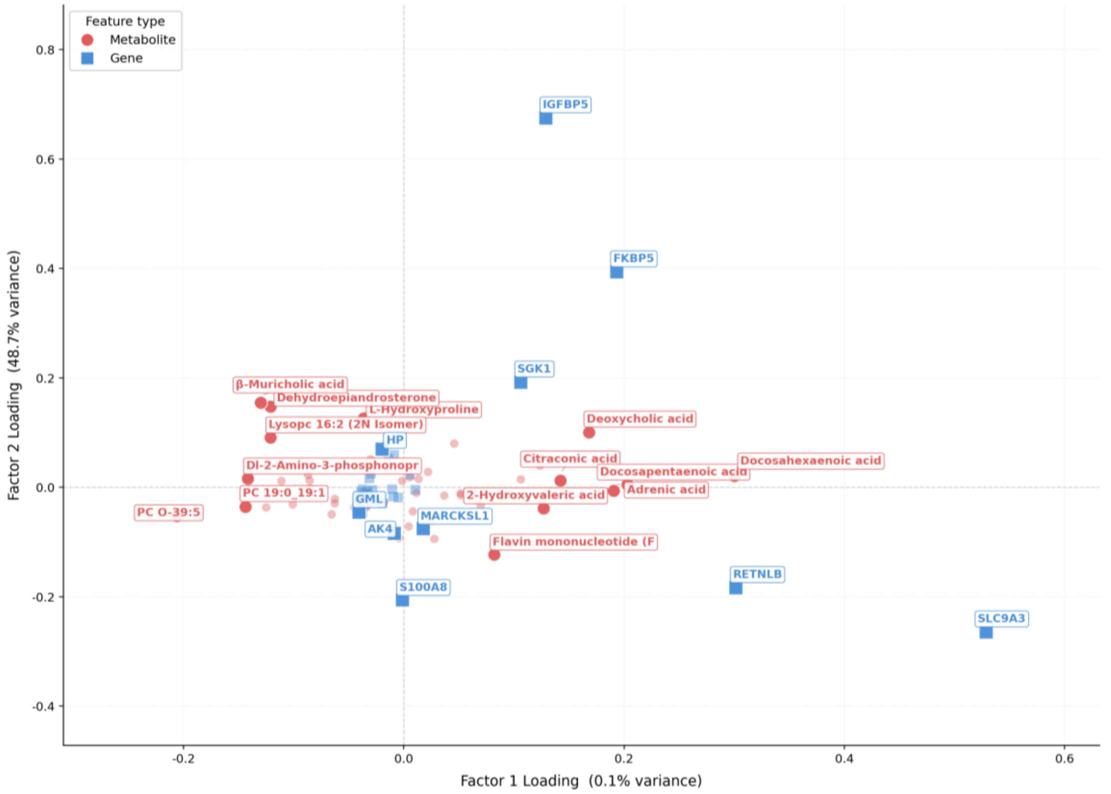

Figure S7. Singular value decomposition identifies dominant axes of integrated multi-omics variation. Loading plot from singular value decomposition (SVD) of the integrated dataset comprising the top 50 metabolites and top 50 differentially expressed genes. Component 1 and Component 2 represent the principal axes of cross-omics variation. Metabolites are shown as red circles and genes as blue squares. Features with the largest loading magnitudes, defined by their Euclidean distance from the origin (top 14 metabolites and top 10 genes), are annotated. Features positioned closer together in the loading space contribute more similarly to the same axes of variation. n=10 samples total (5 mice per group) .
